# Poverty Alleviation Relocation, Fuelwood Consumption and Gender Differences in Human Capital Improvement

**DOI:** 10.3390/ijerph20021637

**Published:** 2023-01-16

**Authors:** Yongtian Zhu, Shigemitsu Shibasaki, Rui Guan, Jin Yu

**Affiliations:** 1 College of Economics and Management, Northwest A&F University, Yangling 712100, China; 2 Graduate School of Agricultural and Life Sciences, University of Tokyo, Bunkyo-ku, Tokyo 113-8657, Japan; 3 School of Politics and Public Administration, Zhengzhou University, Zhengzhou 450001, China

**Keywords:** gender differences, poverty alleviation relocation, fuelwood consumption, human capital, mediation effect, propensity score matching (PSM)

## Abstract

The aim of poverty alleviation relocation is to break the vicious cycle of poverty and ecological degradation. The improvement of human capital, specifically women’s human capital, is important to realize the poverty alleviation and sustainable development of relocated peasant households. Based on the survey data of 902 peasant households in southern Shaanxi in 2020, using the PSM model and the mediation effect test model, this paper explores the impact of participation in relocation on human capital from the perspective of gender differences, and the mediation effect of fuelwood consumption in the effect of participation in relocation on the human capital of peasants with different genders. The results show that firstly, in general, participation in relocation effectively improves the human capital of peasants. Secondly, there are gender differences in the improvement of the human capital of relocated peasants. Compared with male peasants, the health level of female peasants is significantly improved. Finally, fuelwood consumption plays an important mediation role in the impact of participation in relocation on human capital and the mediation role is more significant in improving the human capital of relocated female peasants.

## 1. Introduction

The poverty alleviation relocation policy in China aims to solve the problem that “each place cannot support its own inhabitants” in ecologically fragile areas. According to statistics from the National Development and Reform Commission, by the end of 2020, China had completed the poverty alleviation relocation of more than 9.6 million peasants and built about 35,000 centralized relocation areas and more than 2.66 million relocation houses, playing an important role in China’s poverty reduction process. Participation in relocation reduces the dependence of peasant households on natural resources, alleviates the income gap while improving the income level of peasant households [1,2], and also reduces the poverty vulnerability of peasant households, especially female-headed households [3]. However, due to the damage to social capital caused by the huge changes in the living environment [4], the adaptation difficulties caused by changes in lifestyle, the high cost of relocation, and the economic pressure [5], relocated peasant households still face greater livelihood challenges. The risks of returning to poverty and intergenerational transmission of poverty remain. On this basis, human capital is an important factor to affect the choice of peasants’ livelihood strategies, which is also the core and key to improving income mobility, achieving sustainable livelihoods, and preventing a return to poverty [6,7]. Therefore, accelerating the improvement of the human capital of relocated peasants is of great significance in achieving the policy goal of “stable living and prosperity” and firmly adhering to the bottom line of not returning to impoverishment on a large scale, which has become an important task in the follow-up support phase of relocation.

In the long history of human capital, women’s human capital has gradually received widespread attention and positive recognition from scholars. The research reports of many organizations including the United Nations, government agencies, and non-governmental organizations have put forward policy advocacy related to the improvement of women’s human capital. Women with higher human capital in peasant households not only reduce the risk of returning to poverty but also contribute a gender dividend effect that has many significant and far-reaching positive impacts [8,9]. Firstly, investing in human capital such as women’s health and training can effectively improve women’s well-being, empowerment, and social status [10]. Secondly, women with higher human capital have a higher family decision-making power. They are more inclined to allocate resources to children’s education and households’ nutrition and health [11], which are conducive to enhancing the overall development ability of households and intergenerational occupational mobility of labor forces [12]. Finally, the improvement of women’s human capital in households can effectively release surplus productivity, increase effective labor supply, and promote faster regional economic growth as a whole [13]. Overall, from a gender perspective, more attention needs to be paid to whether poverty relocation alleviation can improve the human capital of female peasants.

Many scholars have explored the changes in the human capital endowment of peasant households brought about by participation in relocation. Wei et al. [14] and Wang et al. [15] pointed out that participation in relocation has effectively improved inter-regional human capital and reduced multidimensional poverty. The higher the human capital, the more likely it is that the livelihood strategy of relocated peasants will be transformed into non-agricultural employment [16]. However, some studies have pointed out that the human capital of the relocated peasants has not increased significantly or even suffered a certain degree of loss. It is embodied in the following two aspects. On one hand, the human capital of peasants such as experience and technology accumulated before relocation is mainly focusing on agricultural and animal husbandry production, which is difficult to meet the requirements of non-agricultural employment after relocation. As a result, participation in relocation brings about a loss of skills transfer and increases the risk of unemployment [17,18,19]. On the other hand, the proportion of poverty of relocated peasants caused by diseases is relatively high. The pressure of increasing income after relocation has shortened the time allocation for family care and leisure within the peasant household and increases the likelihood of deteriorating health of household members [20]. In addition, some scholars have also focused on the impact of poverty alleviation relocation on the lifestyles of peasant households. The spatial changes in the residential location of the relocated peasants have enabled them to achieve leap-forward urbanization in terms of living standards in the short term. However, the lifestyle and social interaction structure have not yet been matched and synchronized, which is not conducive to the stable livelihood of relocated peasants [4].

The influencing factors of human capital are multifaceted and there are many scholars who have pointed to the negative impact of fuelwood consumption on human capital from the perspective of energy utilization. Kyaw et al. [21] and Adjei-Mantey et al. [22] pointed out that excessive fuelwood consumption not only leads to forest destruction and biodiversity loss, increases ecological degradation and natural disasters, but also reduces the food security of farmers’ households, increases air pollution, which brings a greater threat to the lives and health of peasants. At the same time, fuelwood consumption takes up a lot of peasants’ time. This over-reliance on fuelwood consumption has a negative impact on schooling and skills training [23]. In addition, some studies have highlighted gender differences, pointing out that the negative effects brought about by fuelwood consumption are more severe for women than for men [24,25,26,27]. 

On the whole, the existing studies provide a useful reference for analyzing the impact of relocation projects on the human capital of peasant households, but there are still the following deficiencies. Firstly, although scholars have paid attention to the impact of participation in relocation on the human capital of peasant households, they ignore the gender differences characteristics in human capital changes of relocated peasants and pay insufficient attention to the improvement of women’s welfare brought by participation in relocation. Secondly, at present, the analysis of the lifestyle change of the relocated households is still insufficient and few scholars have quantitatively analyzed the changes in the lifestyle of relocated households from the perspective of energy utilization structure. Finally, there is still a lack of deep mechanism analysis of the impact of participation in relocation on human capital.

In view of this, based on the data of 902 peasant households in southern Shaanxi Province in 2020, China, using the propensity score matching (PSM) and the mediation effect test model, the paper tries to solve the following questions: Can poverty alleviation relocation improve the human capital, and whether female peasants can benefit from it or not? Whether fuel consumption is one of the effective ways for relocation project work or not? By solving these questions, this paper can not only provide policy recommendations for follow-up relocation support but also provide references for similar relocation projects in fragile ecological environments around the world. 

## 2. Theoretical Analysis and Research Hypotheses

**Figure 1:**
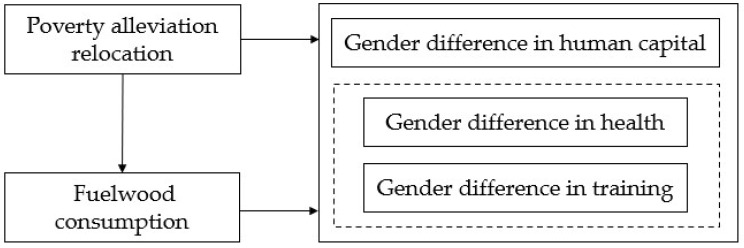
The theoretical framework of the study.

Taking health and training as key manifestations of human capital, this study analyzes the theoretical relationship between poverty alleviation relocation, fuelwood consumption, and human capital from the perspective of gender difference. Figure 1 illustrates the theoretical framework of the study.

### 2.1. Direct Impact of Participation in Relocation on the Human Capital of Peasants

Participation in relocation can directly affect the health and training of peasant households through external resource acquisition and internal expectation promotion. On the one hand, poverty alleviation relocation is a typical poverty alleviation method of resource input and opportunity supply. The grassroots government in the relocated area invests a lot of resources in the construction of medical and health infrastructure [28,29], which improves the level of public medical services in relocation areas and effectively, reduces the risk of disease, and increases the cure rate. At the same time, the grassroots government also actively implements skills training with the core goal of employment, greatly enhancing the opportunities for relocated peasants to receive training. The relative concentration of the relocated population is more conducive to improving the spatial distribution pattern of factors, thereby improving the matching and service efficiency of public resources such as medical care and training to the relocated peasants. As a result, the human capital level of the relocated peasants’ health and training has been improved overall. Moreover, the closer the distance between different relocated sites, the more spatial advantages of resource and opportunity allocation can be exerted, which has a better effect on health and training improvement [30]. In addition, compared with male peasants, female peasants are more inclined to engage in social interactions and improve regional social capital. From which they can draw resources to cope with livelihood risks. [31]. Therefore, female peasants are better adapted to the environment in the relocated areas by accumulating social relationships. This is beneficial for them to obtain medical resources and training opportunities in relocated areas, which effectively improve the human capital.

On the other hand, livelihood pressures such as changes in lifestyles, the rising cost of living, and the decline in agricultural production income urgently require relocated peasants to change their livelihood strategies [14]. Health is the foundation for peasants to engage in all livelihood activities, and training not only efficiently translates into productivity but also improves the information acquisition ability and cognition level of peasants. Therefore, health and training are important foundations and key influencing factors for peasants’ livelihood strategy and non-agricultural transformation [32]. The outcome expectation of obtaining better non-agriculture employment provides an important incentive for relocated households to invest in health and training. In addition, the relocation project weakens the traditional “feminization of agriculture” feature, which not only releases a large number of women surplus labor forces [33] but also changes the traditional gender roles played by women in peasant households. The expectations that female labor forces share the household livelihood pressure and improve the sustainable development ability of peasant households urgently drive them to complete the transition from the agricultural sector to the non-agricultural sector, which in turn raises the realistic demand to improve the health and training of women in relocated peasant households. Therefore, this paper proposes the hypotheses:

**H1:**  Participation in relocation can improve the level of human capital.

**H2:**  There are gender differences in the impact of participation in relocation on human capital, that is, compared with male peasants, the improvement of the human capital of female peasants is bigger.

### 2.2. Indirect Impact of Participation in Relocation on Human Capital of Peasants

In terms of determinants of fuelwood consumption, the energy ladder theory based on income factors perceives a continuous monotonic fuel substitution process as income increases, and ‘inferior’ fuelwood consumption will eventually transit to ‘superior’ modern commercial fuels [34]. However, based on cost, convenience, housing conditions, and other considerations, the energy ladder theory has been refuted by the findings of a growing number of studies [35,36]. Combining the two types of research, participation in relocation can change the fuelwood consumption of peasant households in the following three ways. Firstly, considering the income factor, peasant households have low income before relocation, so they prefer traditional fuelwood energy in energy consumption decisions and lack the ability and motivation to change energy utilization patterns. The relocation project effectively improves the peasant household’s income level [1]. Higher-income levels narrow the constraints on energy access and greatly improve the peasants’ ability to pay for other more efficient energy, which reduces the level of fuel consumption of peasant households. Secondly, the relocation project increases the geographical distance for peasant households to obtain traditional fuelwood, which greatly increases the marginal cost of consuming fuelwood. Correspondingly, the infrastructure of energy supply is gradually improving in relocated areas [37]. Peasant households find it easier to get clean energy sources. So based on the consideration of economic rationality, peasant households will reduce fuelwood consumption by energy substitution. Thirdly, most of the living houses in relocated communities are dominated by buildings. The changes in indoor ventilation conditions and the configuration of modern kitchen facilities are not compatible with traditional fuelwood energy consumption, which reduces the possibility of fuel stacking and the inertia of energy utilization [36]. The urbanization community’s management mode also urgently requires the relocated households to change their lifestyles and adopt healthy and civilized ways of energy utilization. Summarizing the above analysis, relocation project greatly reduces the fuelwood consumption of households.

Reduction in fuelwood consumption can improve training through time reallocation and improve health through air pollution alleviation and malnutrition improvement. Considering the fact that women are mainly responsible for fuelwood collection and utilization [24], the reduction in fuelwood consumption is more conducive to improving the training and health of women. Specifically, the time constraint is a loss of time due to the double inefficiency of fuelwood collection and fuelwood utilization. The reduction in fuelwood consumption will significantly save time for those responsible for energy use and collection, mainly women in peasant households. The time burden of collecting fuelwood on women and girls is well recognized by the United Nations in its focus on gender education and equality efforts [27]. At the same time, this conclusion has also been confirmed by numerous scholarly studies. Clancy et al. [25] pointed out that due to the substitution of fuelwood consumption, women’s time saved can range from 3 to 20 h per week for firewood collection cooking. On this basis, guided by the classical theory of time allocation proposed by Gronau [38], time substitution for the reduction of fuelwood consumption is conducive to women allocating more time to participate in training to seek non-agricultural employment opportunities [39].

From the perspective of health constraints, on one hand, the combustion of fuelwood will produce a large number of toxic gases such as CO and PM, as well as particulate matter [40]. This not only causes indoor and outdoor air pollution but also easily leads to respiratory diseases, cardiovascular disease, and so on, which greatly endanger health. This issue has been widely recognized and has been identified as one of the top four global health problems by the World Health Organization [41]. On the other hand, due to the lack of access to other clean energy sources, fuelwood consumption can also affect nutrition by daily cooking frequency and meal cooked type, especially in poor areas. A study by Baron and Kenny based on the Kenyan pointed out that peasants generally adopt two meals a day or one meal and choose a single food with shorter cooking times to cope with energy shortages and low energy utilization rates [42]. This can cause health damage due to nutritional imbalance. Therefore, the reduction in fuelwood consumption can alleviate air pollution and malnutrition, thereby increasing health capital. Based on the above viewpoints, this paper proposes the following hypotheses:

**H3:**  Fuelwood consumption plays a mediation role in the impact of participation in relocation on human capital, that is, participation in relocation can improve the level of human capital by reducing fuelwood consumption.

**H3a:**  Participating in the relocation can reduce the fuelwood consumption of peasant households.

**H3b:**  The reduction of fuelwood consumption can effectively improve the human capital of peasant households.

**H4:**  Compared with the male peasants, the reduction of firewood consumption plays a greater role in improving the human capital of female relocated peasants.

## 3. Methodology

### 3.1. Study Area

The data in this paper come from a farmer survey conducted in Ankang city and Shangluo city in southern Shaanxi Province, China, in December 2020. Located in the Qinba Mountains, the southern Shaanxi region is a typical ecologically fragile area in China, with abundant forestry resources and species resources. However, the area has complex terrain and landform, backward infrastructure construction, frequent natural disasters such as landslides and debris flows, and insufficient resource-carrying capacity. For a long time, the area exists in a vicious circle between the destruction of forest resources caused by unscrupulous human activities, environmental pollution, ecological disasters, and poverty of peasants, which is one of the concentrated and contiguous poverty-stricken areas in China in 2011. In order to ensure the safety of local people’s lives and property, the Shaanxi Province government launched a relocation project involving 2.4 million peasants in 2011, which has achieved remarkable results in poverty reduction and ecological protection. Then, in the period of targeted poverty alleviation that began in 2016, the Shaanxi Province government followed the “Five-pronged Poverty Alleviation Measures” Project and accelerated the implementation of the poverty alleviation relocation from a new starting point. In the nearly ten years since 2011, as a typical area for the implementation of poverty alleviation relocation in China, Shaanxi Province had completed the relocation tasks of more than 2 million peasants.

For different types of peasant households, by providing free public housing or financial compensation for housing, relocation projects promote the relocation of poor households from remote and vulnerable areas to places with more non-agricultural employment opportunities and better living and housing conditions. It has several important characteristics. Firstly, participation in relocation is a voluntary choice and centralized relocation is the main relocation type. Secondly, the relocation destination is mainly nearby towns or counties instead of big cities. Thirdly, the relocation project starts earlier. Finally, the core of the relocation project aims to achieve sustainable development of peasant households. Therefore, multidimensional support for relocated peasants has always been an important content of the relocation project. Among them, reasonably allocated medical and public health resources, continuously improved medical security policies, free and precise vocational skills training, and employment assistance provide important opportunities for relocated peasants to improve their health and training levels.

Taking into account the implementation of the relocation policy and the economic development of different counties in Shaanxi Province, a total of 4 counties including Danfeng county, Zhen’an county, Baihe county, and Hanbin district are selected as the survey areas. Among them, Danfeng county and Zhen’an county belong to Shangluo city, while Baihe county and Hanbin district belong to Ankang city. The research group adopts the stratified random sampling method to select 3 towns in each county, 2–3 villages in each town, and 20–50 households in each village for household investigation and interview. The survey questionnaire includes basic information such as individual and household characteristics, relocation characteristics, production and operation characteristics, energy utilization characteristics, and livelihood capital in different dimensions. A total of 931 questionnaires were returned in this survey. Since this paper explores the impact of participation in relocation on human capital from the perspective of gender, in the process of data cleaning, the questionnaires with incomplete gender structure of the household and abnormal data were deleted. A total of 902 effective sample data were finally obtained. Among them, 692 households have participated in the relocation project.

### 3.2. Model Specification

#### 3.2.1. Propensity Score Matching (PSM) Model

The basic model (OLS) of the impact of relocation on human capital is constructed as follows:

(1) $$Y_{i}=\beta_{0}+\beta_{1}R_{i}+\beta_{2}X_{i}+e_{i}$$

In Equation (1), $Y_{i}$ is the human capital of peasant household i; $R_{i}$ is a dummy variable of whether household i participates in the relocation or not, $R_{i}=1$ means that the household has participated in the relocation and enters the treatment group, and $R_{i}=0$ means that the household has not participated in the relocation and enters the control group; $X_{i}$ is the control variable; $\beta_{0}$ is the constant term; $\beta_{1}$ and $\beta_{2}$ are the estimated coefficients; and $e_{i}$ is the random error term.

Considering that participation in relocation is a self-selected behavior, whether or not peasant households participate in relocation will be affected by factors such as the households’ characteristics of the original housing, economic situation, and capital endowment. In particular, peasant households who originally lived in high mountains or deep slopes, with younger household heads and higher levels of non-agricultural livelihoods, were more inclined to participate in relocation [43]. There is a certain sample selection bias. Therefore, adopting the traditional OLS model may affect the identification effect, and cannot accurately identify the policy effect of participation in relocation. By constructing a “counterfactual” analysis framework, PSM can find the control group that is most similar to the treatment group and eliminate the sample selection bias to the greatest extent, thus effectively solving the problem of endogeneity. Therefore, this paper adopts PSM to further estimate the treatment effect of participating in relocation.

Firstly, the logit model is used to estimate the propensity score matching p(X), which is the conditional probability of sample peasants participating in the relocation.

(2) $$Y_{i}p\left(X\right)=\mathrm{Pr}\left(R=1|X\right)=E\left(R=0|X\right)$$

Secondly, according to the propensity score value, three matching methods of nearest-neighbor matching (NNM), caliper matching (CM), and kernel-based matching (KBM) are used to match the samples of the treatment group (*R* = 1) and the control group (*R* = 0).

Finally, calculating the average treatment effect on the treated (ATT), that is, the impact of participation in relocation on human capital:

(3) $$ATT=E\left(Y_{1i}-Y_{0i}|R_{i}=1\right)=E\left[E\{Y_{1i}-Y_{0i}|R_{i}=1,p\left(X_{i}\right)\}\right]\;=E\left[E\{Y_{1i}|R_{i}=1,p\left(X_{i}\right)\}-E\{Y_{0i}|R_{i}=0,p\left(X_{i}\right)\}|R_{i}=1\right]$$

In Equation (3),$\;E\{Y_{1i}|R_{i}=1,p\left(X_{i}\right)\}$ is the human capital of the relocated peasants that can be directly observed, $E\{Y_{0i}|R_{i}=0,p\left(X_{i}\right)\}$ is the “counterfactual” estimate of the human capital of non-relocated peasants by propensity score matching.

#### 3.2.2. Mediation Effect Test

In order to test the mediation effect of fuelwood consumption on the impact of participation in relocation on human capital, this paper uses the stepwise regression analysis proposed by Baron and Kenny [44] and establishes the following model based on the basic model (OLS):

(4) $$M_{i}=a_{1}R_{i}+a_{2}X_{i}+e_{2i}$$

(5) $$Y_{i}=d_{1}R_{i}+d_{2}M_{i}+d_{3}X_{i}+e_{3i}$$

In Equations (4) and (5), $\;Y_{i}$ is the human capital of the peasant households *i*, $R_{i}$ and $X_{i}$ are the same as Equation (1), which are the participation in relocation and control variables, respectively. $M_{i}$ is the moderator which is fuelwood consumption. $a_{1}$ is the effect of participation in relocation on moderator. $d_{1}$ is the direct effect of participation in relocation on human capital after controlling the influence of moderator. Substitute Equation (4) into Equation (5) to obtain the mediation effect $d_{2}a_{1}$, that is, the effect of participation in relocation on human capital through moderator. $e_{1i}$, $e_{2i}$, and $e_{3i}$ are random error terms. If $\beta_{1}$ in Equation (1), $a_{1}$ in Equation (4), and $d_{2}$ in Equation (5) are all significant, it indicates that moderator *M* has played a significant mediation effect.

### 3.3. Variable

#### 3.3.1. Dependent Variables

The measurement of human capital in academic research is relatively mature. Its external performance is generally defined as the knowledge, skills, and qualities possessed by individuals [45], measured by variables such as education, health, and training. However, on the one hand, it takes a long time to improve the education level of the peasants, and it takes necessary investment and accumulation to transform education into the improvement of productivity. Furthermore, formal education is difficult to fully reflect the quality of human capital, which may underestimate the economic returns of human capital [46]. On the other hand, health is the basis of all factors concerning human capital [47] which has an important impact on the achievement of a sustainable livelihood by peasants. Training is an important way to invest in human capital for disadvantaged peasants in the labor market. The improvements in health and training are easier and it is more efficient to translate into family development capacity, productivity, and regional economic development [48,49].

Based on this, this paper uses the two variables of per capita health and the number of peasants trained to measure the human capital. Considering that air pollution caused by fuelwood consumption is associated with various diseases such as respiratory infections, cardiovascular disease, neurological disorders, chronic inflammation low birth weight, and cataracts. Furthermore, the energy utilization pattern of fuelwood consumption can also affect health by malnutrition caused by cooking frequency and meal cooked type. Therefore, the overall health level of peasants is used to measure. At the same time, this paper also distinguishes it from the gender perspective and constructs four gender human capital variables including the per capita health of women, the per capita health of men, the number of women trained, and the number of men trained.

#### 3.3.2. Core Independent Variable

Participate in relocation. This variable is a dummy variable, which is obtained based on the responses of the surveyed households to the question “Have you participated in poverty alleviation relocation?”. If the peasant households participated in the relocation, the value is 1; otherwise, it is 0.

#### 3.3.3. Moderator

Per capita fuelwood consumption. In this paper, the variable is defined as the per capita weight of fuelwood consumed by households in lighting, cooking, heating, and other living activities in one year [50]. The amount of fuelwood consumed includes the sum of all sources, including forest, agricultural land, and purchases.

#### 3.3.4. Matching and Control Variables

According to the research of scholars, the matching variables should try to select household characteristics variables related to participation in relocation, fuelwood consumption, and human capital at the same time [51]. Therefore, in the PSM model, this paper selects five household characteristic variables, including household size, village cadres, number of children, number of elderly, and household head age. In terms of household production and living condition variables, a total of 8 variables including forest area, land area, livelihood non-agricultural, savings, future development expectation, relatives and friends in the city, formal borrowing difficulty, and informal borrowing difficulty were selected. Meanwhile, in the OLS model and mediation effect test, two control variables, Shangluo city and distance to county, were introduced to control regional heterogeneity.

The definitions of the variables in this paper are shown in Table 1.

Before the regression analysis, this paper compares the differences in the main variables between the relocated and non-relocated peasant households and conducts the t-test for the significance of the mean difference. As shown in Table 1, except for the four variables of village cadres, the number of the elderly, savings and informal borrowing difficulty, the remaining control variables and human capital variables between relocated and non-relocated households are significantly different. This further verifies that there is a certain endogeneity in relocation behavior, and sample selection bias needs to be considered when exploring the impact of the relocation project.

## 4. Estimated Results and Analysis

### 4.1. Logit Model on Determinants of Participation in Relocation

As can be seen from Table 2, in terms of household characteristics, household head age had a significant negative impact on participation in relocation at the significant level of 5%. The older the household head, the deeper the plot of “Love one’s home and one’s land”, so the elder is unwilling to change his original residential location [52], and the possibility of participating in relocation is low.

In terms of household production and living conditions, firstly, future development expectations had a significant positive impact on relocation behavior. Participation in relocation is a household decision with the coexistence of opportunities and risks [53], and better future development expectation greatly improves the level of endogenous power of peasants to participate in relocation. Secondly, forest area had a significant positive effect on participation in relocation, while land area had a significant negative effect. Forests in southern Shaanxi are mainly ecological forests. More forest area and less land area both mean lower agricultural income and less dependence on natural resources, driving the relocation behavior of peasant households. Finally, relatives and friends in the city, and formal borrowing difficulty significantly negatively affected the relocation behavior of peasant households. Accumulating more social relations, obtaining more developing resources, and improving household savings levels through relocation are important motivations for peasant households to participate in relocation. The easier access to financial support such as loans has not only effectively reduced peasants’ expectations of livelihood risks after the relocation, but also enhanced peasants’ ability to resist risks, thus improving the possibility of households participating in the relocation.

### 4.2. Estimated Results of the Treatment Effect of Participation in Relocation on Human Capital

Table 3 presents the estimated results for the impact of participating in relocation on human capital under different genders using the OLS model and PSM with 3 different matching methods. Overall, participation in relocation significantly improves per capita health and the number of peasants trained. To be specific, when NNM, CM, and KBM were used, ATT on health were 0.188, 0.211, and 0.208, respectively, which were all significantly positive at the 5% significant level. The ATT on training were 0.120, 0.114, and 0.109, respectively, which were all significantly positive at the 1% significant level. The relocation project not only allows farmers to access the various development resources and opportunities needed to improve human capital but also increases their willingness and demand for human capital accumulation. As a result, participating in relocation has effectively improved the level of human capital, which laid a solid foundation for the relocated peasant households to break the intergenerational transmission of poverty and achieve the upward flow of income. Hypothesis 1 has been verified.

From the estimated results of human capital in different genders, in terms of health, the results of the OLS and PSM both show that the per capita health of women in the relocated households has improved significantly. The per capita health level of men has also improved to some extent, but the PSM estimated results are not significant. In terms of the number of peasants trained, participation in relocation has a significant positive impact on different genders, and the ATT of women is lower than that of men. In summary, hypothesis 2 is only partially verified.

### 4.3. Matching Quality

In order to ensure the matching quality, the following three metrics should be evaluated: (1) the degree to which the overall bias is reduced due to matching; (2) the common support area between the treatment group and the control group; and (3) balance test between the treatment group and the control group.

Firstly, before matching, most variables have large deviations and biases. After matching, the standardized deviation of all variables was significantly reduced. Except that the standardized deviation of the number of children (% bias) was 10.2%, and the standardized deviation of other variables is less than 10%.

Secondly, the distribution of predicted propensity scores for the relocation and non-relocation households showed a large common support of propensity scores. The sample loss of the three matching methods was similar. Taking NNM as an example, there were only 3 observations of support in the control group and 40 observations of support in the treatment group.

Finally, in terms of the balance test, as shown in Table 4, LR chi2 and Mean Bias of the whole observations after matching were significantly lower than those before matching. The B-stat dropped below 25%, the Pseudo R^2^ was almost 0, and the R-stat was closer to 1.

This result verifies the good sample matching quality which increases the confidence in the matching estimates. Different matching methods significantly all reduce the difference in matching variables between the treatment group and the control group, which minimizes the sample selection bias.

### 4.4. Estimated Results of Mediation Effect

Table 5 shows the estimated results of the mediation effect of fuelwood consumption. According to the stepwise regression analysis, first of all, the previous part has explored the significant positive impact of participation in relocation on human capital, that is, $\beta_{1}$ was significant, so further mediation effect analysis can be carried out. Second, as shown in the first row of Table 5, participation in relocation had a significantly negative impact on fuelwood consumption at the significant level of 1%, that is, $a_{1}$ was significant. This shows that participating in relocation has effectively reduced the fuelwood consumption of peasant households, which plays a positive role in improving energy utilization efficiency and protecting the ecological environment. Hypothesis 3a is verified.

Rows 2 and 3 in Table 5 present the estimated results of the effect of participation in relocation and fuelwood consumption on human capital. After controlling the effect of participation in relocation, the coefficient of the effect of fuelwood consumption on per capita health and number of peasants trained were both significantly negative at the 5% significant level. Hypothesis 3b is verified. This suggests that fuelwood consumption plays a significant mediation effect in the effect of participation in relocation on human capital. Participation in the relocation can effectively reduce fuelwood consumption and thus improve the level of peasants’ health and training. On the one hand, improved housing conditions, higher income, and the increased marginal cost of fuelwood consumption are important factors for the reduction of fuelwood consumption of relocated peasants. On the other hand, fuelwood consumption can improve the human capital of peasants by alleviating time constraints, reducing the likelihood of disease, and improving malnutrition. Therefore, hypothesis 3 is verified.

Considering that the impact of fuelwood consumption on the human capital of different genders may have heterogeneity characteristics, this section further explores the mediation effect of fuelwood consumption in the impact of participation in relocation on the human capital of different genders. As shown in rows 4 and 5 of Table 5, including participation in relocation and fuelwood consumption into the regression of the effect on the human capital of female peasants, the two coefficients of fuelwood consumption were both significantly negative. That is, after controlling for the effect of participation in relocation, the reduction in fuelwood consumption significantly increases the health and training level of women and plays a significant mediation effect in the effect of relocation on increasing women’s human capital. Rows 6 and 7 in Table 5 show the estimated results that participation in relocation and fuelwood consumption are jointly included in the regression of the effect on the human capital of male peasants. The two coefficients of the fuelwood consumption did not significant, so the mediation role of fuelwood consumption in the effect of participation in relocation on the human capital of male peasants was not significant. To sum up, compared with men, fuelwood consumption plays a greater mediation effect on the improvement of the human capital of relocated female peasants. Due to differences in family responsibilities, the change in fuelwood utilization brought by the relocation project not only more effectively increases the available time for female peasants to spend in training, but also improves their health by reducing the possibility of direct exposure to polluted air caused by fuelwood burning. Hypothesis 4 is verified.

## 5. Discussion

From the above results, hypotheses 1, 3, and 4 proposed in this paper are well verified, while hypothesis 2 is partially verified.

In terms of hypotheses 1 and 2, this study finds that poverty alleviation relocation can effectively improve the human capital of peasants, which is basically consistent with the existing research conclusions and verifies the rationality and effectiveness of the implementation of relocation projects. Unlike the existing research, by introducing four gender human capital variables, this paper focuses on the differential impact of the relocation project from a gender perspective. This is a profound exploration of the policy effects of the relocation project and a deep focus on disadvantaged female peasants, which provide a new idea for the study of poverty alleviation relocation and have important theoretical significance. The results of this paper confirm that the health and training levels of female relocated peasants have been significantly improved, which will effectively improve the welfare and empowerment of women. This is not only important for consolidating the poverty alleviation achievements, but also will play a role in the gender dividend and have significant and long-term impacts in many dimensions.

It is inconsistent with hypothesis 2 that although the training participation of female peasants has been improved, the level of improvement is lower than that of men and still has room for improvement. A possible reason is that the resource allocation mode of peasant households is characterized by economic rationality and distribution order, aiming at maximizing economic benefits, and gives priority to meeting the needs of the most important members with the highest labor productivity in households [54]. Compared with men, women’s time consumption of housework activities and caring are hardly regarded as having economic value. So, they are in a disadvantaged position and lower order in resource allocation and have fewer opportunities to obtain development resources. The training resources input by the government are scarcer for households and more valuable to non-agricultural employment, so training resources are more likely to be preferentially acquired by male peasants.

In terms of hypotheses 3 and 4, fuelwood consumption can play an important mediation role in the impact of relocation on human capital, which is particularly beneficial to improving the human capital of female relocated peasants. The adverse effects of fuelwood consumption on health and training have been tested in many studies. On this basis, this paper innovatively identifies the important role of reducing fuelwood consumption plays in the functioning of the relocation project, which provides important policy implications for effectively consolidating poverty alleviation outcomes and improving the human capital of relocated households. In particular, in the context of China’s active promotion of rural renewable energy, seizing the policy opportunity to replace fuelwood consumption by actively developing new energy sources in the relocation areas will achieve a win–win situation for both rural revitalization and green and low-carbon energy transition.

The theoretical contributions of this study are as follows: (1) Unlike studies that focus on the impact of poverty alleviation relocation on human capital by treating households as a homogeneous whole, this paper innovatively discusses gender differences in the effects of the relocation project, especially focusing on the improvement of the human capital of women by participating in relocation, which is a further deeply exploration and an important theoretical supplement of existing studies. (2) This paper explores the impact of poverty alleviation relocation on fuelwood consumption from the perspective of energy utilization, which is an important addition to the empirical study on the transformation of relocated farmers’ lifestyles. (3) By including fuelwood consumption, this paper constructs a theoretical analysis paradigm, which is not only a reference for studies related to relocation and human capital but also expands the research on the mechanism of improving the human capital of relocated peasants from the perspective of energy utilization.

In addition, this paper has some deficiencies, which can be addressed in future studies. Specifically, (1) this paper measures human capital using health and training. Future studies can further enrich the indicator for measuring human capital and explore the relationship between relocation projects and human capital. (2) The relocation project has brought many changes to the peasants’ lifestyle, of which fuel consumption is only one part. Future research can provide empirical evidence for the life change of relocated peasants from the aspects of the transformation of pension mode and social model in relocated communities and analyze its impact on sustainable livelihoods. (3) There may be heterogeneous characteristics of the effect of poverty alleviation relocation on human capital by different relocation types, relocation distances, and follow-up support types for peasant households. Future studies can further add indicators related to relocation policies for analysis to put forward feasible suggestions for improving follow-up relocation support policies in other dimensions.

## 6. Conclusions and Policy Recommendations

From the perspective of gender differences, this paper explores the impact of participation in relocation on human capital, and further tests the mediation effect of fuelwood consumption in the effect of participation in relocation on human capital. The research conclusions are as follows:

Firstly, participation in relocation effectively improves human capital, including health and training, and plays an important role in consolidating the results of poverty alleviation and achieving sustainable household livelihoods.

Secondly, there are heterogeneous characteristics of the effect of relocation projects on the human capital of peasant households with different genders. On the one hand, compared with male peasants, participation in relocation significantly increases the health of female peasants, which effectively improves the welfare level of women. On the other hand, although the training participation of relocated peasants of different genders increases significantly, among female peasants there is still room for improvement.

Thirdly, participation in relocation has effectively reduced fuelwood consumption and changed the energy utilization structure. At the same time, the reduction of fuelwood consumption has a positive impact on improving the human capital of peasants. Therefore, fuelwood consumption plays an important mediation role in the impact of participation in relocation on human capital. Compared with relocated male peasants, the mediation role is more significant in improving the human capital of relocated female peasants.

Based on the above conclusions, this paper provides the following policy implications for improving human capital. Firstly, relying on poverty alleviation relocation, peasant households have achieved an increase in human capital. On the one hand, the government should continue to strengthen the follow-up support for relocation. Not only improving the practicality and coverage of training, but also accelerating the match of the health insurance system, and gradually improving the level of medical services in the relocated area. On the other hand, policy dependency and short-term effects should be avoided. By strengthening employment support and improving the regional labor market environment, the governments should improve the return on investment of the human capital of relocated peasants and realize a virtuous cycle of human capital accumulation. Secondly, considering that the training status of relocated female peasants still has room for improvement, the governments in relocated areas should not only improve the status of women by actively organizing cultural activities and advocating reasonable gender equality concepts but also strengthen the inclination of resources such as labor skill training to women. For example, targeting women, providing various learning and training methods, granting training subsidies, and implementing incentive policies for acquiring certificates of technical skills. Thirdly, based on the important role played by reducing fuelwood consumption, on the one hand, relying on the geographical characteristics of the relocated areas, the governments should vigorously encourage the development of renewable energy industries, accelerate the construction and comprehensive use of new energy represented by photovoltaics, increase the coverage of distributed photovoltaics on the roofs in the relocation area to optimize the energy consumption structure of peasants. On the other hand, while strengthening the regulation and punishment of illegal fuelwood consumption, actively promoting and publicizing the technologies and related supporting equipment of renewable energy sources.

## Figures and Tables

**Table 1:** Variable definitions and descriptive statistics.

Variables		Description	All Mean	Treatment Group Mean	Control Group Mean	T (*p*-Value)
Dependent variables						
	Per capita health	Per capita health of household. Bad = 1; poor = 2; general = 3; good = 4; very good = 5 data	4.042	4.106	3.829	−0.277 ***
	Per capita health of women	Per capita health of women in household	3.995	4.063	3.771	−0.292 ***
	Per capita health of men	Per capita health of men in household	4.062	4.122	3.865	−0.257 ***
	Number of peasants trained	Number of peasants that have participated in labor skills training	0.239	0.269	0.143	−0.126 ***
	Number of women trained	Number of women that have participated in labor skills training	0.047	0.055	0.019	−0.036 **
	Number of men trained	Number of men that have participated in labor skills training	0.193	0.214	0.124	−0.090 ***
Moderator						
	Per capita fuelwood consumption	Per capita weight of fuelwood consumption by households in a year for lighting, cooking, heating, and other activities, take the natural log	4.013	3.670	5.144	1.475 ***
Matching and control variables						
	Household size	Total number of people in the household	4.306	4.357	4.138	−0.218 **
	Village cadres	Whether there are village cadres in households? Yes = 1, no = 0	0.065	0.059	0.086	0.065
	Number of children	Number of persons aged 18 and below in the household	0.956	1.004	0.795	−0.209 ***
	Number of the elderly	Number of persons over 65 years old in the household	0.340	0.334	0.362	0.028
	Household head age	29 years old and below = 1, 30–39 years old = 2, 40–49 years old = 3, 50–59 years old = 4, 60 years old and above = 5	3.702	3.642	3.900	0.258 ***
	Forest area	Currently actual operating forest area (mu, the Chinese version of acre, which is commonly 666.7 square meters)	10.955	13.232	3.449	−9.784 ***
	Land area	Currently actual operating land area (mu, the Chinese version of acre, which is commonly 666.7 square meters)	1.187	0.996	1.815	0.819 ***
	Livelihood non-agricultural	Proportion of non-agricultural income to total income (%)	0.856	0.866	0.824	−0.042 **
	Savings	The current deposit amount of the household (10,000 CNY); [0,1) = 1; [1,3) = 2; [3,5) = 3; [5,10) = 4; [10,+∞) = 5	2.572	2.522	2.738	0.216
	Future development expectation	Bad = 1; poor = 2; general = 3; good = 4; very good = 5	3.975	4.009	3.862	−0.147 *
	Relatives and friends in city	Total number of relatives and friends in city	3.182	2.978	3.852	0.874 **
	Formal borrowing difficulty	Easy = 1, easier = 2, general = 3, hard = 4, very hard = 5	2.369	2.285	2.648	0.353 ***
	Informal borrowing difficulty	Easy = 1, easier = 2, general = 3, hard = 4, very hard = 5	2.399	2.363	2.519	0.143
	Shangluo city	Yes = 1, no = 0	0.509	0.506	0.519	0.013
	Distance to county	Distance from the peasant household’s location to the nearest county (km); [0,10) = 1; [10,20) = 2; [20,30) = 3; [30,40) = 4; [40,+∞) = 5	3.415	3.382	3.524	0.142

Note: ***, **, * denote significance at 1% level, 5% level, and 10% level, respectively.

**Table 2:** Logit model results of determinants of participation in relocation.

Variables	Coefficient	Clustered S. E.	Z-stat	Confidence Interval
Household size	0.0545	0.095	0.58	−0.131, 0.240
Village cadres	−0.466	0.326	−1.43	−1.105, 0.172
Number of children	0.146	0.136	1.07	−0.121, 0.414
Number of the elderly	−0.114	0.147	−0.77	−0.402, 0.175
Household head age	−0.225 **	0.095	−2.36	−0.412, −0.038
Forest area	0.034 ***	0.010	3.52	0.015, 0.053
Land area	−0.142 ***	0.040	−3.57	−0.220, −0.064
Livelihood non-agricultural	−0.099	0.365	−0.27	−0.814, 0.616
Savings	−0.178 **	0.073	−2.45	−0.321, −0.036
Relatives and friends in the city	−0.051 ***	0.018	−2.90	−0.086, −0.017
Future development expectation	0.248 **	0.103	2.41	0.046, 0.449
Formal borrowing difficulty	−0.284 **	0.101	−2.82	−0.482, −0.087
Informal borrowing difficulty	0.050	0.105	0.48	−0.156, 0.256
Constant	2.061 ***	0.698	2.95	0.694, 3.428
LR chi2(13)	94.46			
Prob > chi2	0.000			

Note: *** and ** denote significance at 1% level and 5% level, respectively.

**Table 3:** Estimated results of PSM model with different matching methods.

Variables	OLS	Unmatched	NNM (N = 4)	CM (C = 0.010)	KBM (Bandwidth = 0.06)
	ATT	ATT	ATT	ATT	ATT
Per capita health	0.188 ***	0.277 ***	0.188 **	0.211 **	0.208 **
	(0.062)	(0.064)	(0.092)	(0.090)	(0.084)
Per capita health of women	0.201 ***	0.292 ***	0.215 **	0.218 **	0.212 **
	(0.071)	(0.072)	(0.102)	(0.100)	(0.093)
Per capita health of men	0.169 **	0.257 ***	0.141	0.180	0.181
	(0.077)	(0.079)	(0.110)	(0.108)	(0.100)
Number of peasants trained	0.154 ***	0.126 ***	0.120 ***	0.114 ***	0.109 ***
	(0.040)	(0.039)	(0.042)	(0.041)	(0.039)
Number of women trained	0.039 **	0.036 **	0.042 **	0.042 **	0.041 **
	(0.018)	(0.017)	(0.019)	(0.018)	(0.017)
Number of men trained	0.115 ***	0.090 ***	0.077 **	0.071 ***	0.068 **
	(0.035)	(0.034)	(0.037)	(0.036)	(0.034)

Note: NNM, nearest-neighbor matching method; KBM, kernel-based matching method; CM, radius matching method. ATT, average treatment effect on the treated. *** and ** indicate significant levels at 1% and 5%, respectively. Bootstrap standard errors are in parentheses.

**Table 4:** Balance tests before and after matching.

Matching Methods	Pseudo R^2^	LR chi2	Mean Bias	B-stat	R-stat
Before matching	0.096	94.82	19.9	68.7 *	2.28 *
NNM	0.006	11.15	5.6	18.5	1.10
KMB	0.006	10.61	4.8	18.2	1.07
CM	0.005	9.57	3.8	17.2	1.10

Note: NNM, the nearest-neighbor matching method; KBM, kernel-based matching method; CM, radius matching method. * indicates significant levels at 10%.

**Table 5:** Estimated results of mediation effect of fuelwood consumption.

Gender	Paths	Relocation→Fuelwood Consumption→Health		Relocation→Fuelwood Consumption→Peasants Trained	
		Coefficient	S.E.	Coefficient	S.E.
All	Impact of relocation on fuelwood con-sumption ($a_{1}$)	−1.083 ***	0.251	−1.083 ***	0.251
	Impact of fuelwood consumption on human capital ($d_{2}$)	−0.021 **	0.008	−0.012 **	0.005
	Impact of relocation on human capital ($d_{1}$)	0.166 ***	0.062	0.141 ***	0.041
Women	Impact of fuelwood consumption on human capital ($d_{2}$)	−0.027 ***	0.009	−0.007 ***	0.002
	Impact of relocation on human capital ($d_{1}$)	0.172 **	0.072	0.031 *	0.018
Men	Impact of fuelwood consumption on human capital ($d_{2}$)	−0.017	0.010	−0.005	0.005
	Impact of relocation on human capital ($d_{1}$)	0.151 *	0.078	0.110 ***	0.036

Note: *, **, and *** indicate significant levels at 10, 5, and 1%, respectively. S.E. indicates standard error.

## Data Availability

The data presented in this study are available on request from the corresponding author.
